# Competitive Replacement of Invasive Congeners May Relax Impact on Native Species: Interactions among Zebra, Quagga, and Native Unionid Mussels

**DOI:** 10.1371/journal.pone.0114926

**Published:** 2014-12-09

**Authors:** Lyubov E. Burlakova, Brianne L. Tulumello, Alexander Y. Karatayev, Robert A. Krebs, Donald W. Schloesser, Wendy L. Paterson, Traci A. Griffith, Mariah W. Scott, Todd Crail, David T. Zanatta

**Affiliations:** 1 Great Lakes Center, SUNY Buffalo State, Buffalo, New York, United States of America; 2 The Research Foundation of The State University of New York, SUNY Buffalo State, Office of Sponsored Programs, Buffalo, New York, United States of America; 3 Cleveland State University, Department of Biological, Geological, and Environmental Sciences, Cleveland, Ohio, United States of America; 4 U.S. Geological Survey, Great Lakes Science Center, Ann Arbor, Michigan, United States of America; 5 Central Michigan University, Institute for Great Lakes Research, Biology Department, Mount Pleasant, Michigan, United States of America; 6 University of Toledo, Department of Environmental Science, Lake Erie Center, Toledo, Ohio, United States of America; Gettysburg College, United States of America

## Abstract

Determining when and where the ecological impacts of invasive species will be most detrimental and whether the effects of multiple invaders will be superadditive, or subadditive, is critical for developing global management priorities to protect native species in advance of future invasions. Over the past century, the decline of freshwater bivalves of the family Unionidae has been greatly accelerated by the invasion of *Dreissena*. The purpose of this study was to evaluate the current infestation rates of unionids by zebra (*Dreissena polymorpha*) and quagga (*D. rostriformis bugensis*) mussels in the lower Great Lakes region 25 years after they nearly extirpated native unionids. In 2011–2012, we collected infestation data for over 4000 unionids from 26 species at 198 nearshore sites in lakes Erie, Ontario, and St. Clair, the Detroit River, and inland Michigan lakes and compared those results to studies from the early 1990s. We found that the frequency of unionid infestation by *Dreissena* recently declined, and the number of dreissenids attached to unionids in the lower Great Lakes has fallen almost ten-fold since the early 1990s. We also found that the rate of infestation depends on the dominant *Dreissena* species in the lake: zebra mussels infested unionids much more often and in greater numbers. Consequently, the proportion of infested unionids, as well as the number and weight of attached dreissenids were lower in waterbodies dominated by quagga mussels. This is the first large-scale systematic study that revealed how minor differences between two taxonomically and functionally related invaders may have large consequences for native communities they invade.

## Introduction

Positive interactions among invaders that may enhance their probability of survival and thus cause a greater impact on the recipient community [1] received much attention in invasion biology. However, the opposite phenomenon when two invaders impact each other negatively (“invasional interference”) and thus reduce invasion success and potentially the overall effect on the native ecosystem, has been overlooked [2]. Determining whether the effects of multiple invaders will be superadditive, additive, or subadditive, has critical implications for the Holy Grail of invasion biology – predicting impacts of invasive species [3], and for prioritization of management efforts [4].

Freshwater bivalves of the family Unionidae (unionids) have been declining over the past century and are considered to be among the most endangered groups of animals in North America [5],[6]. The main drivers of this decline have been habitat destruction, deterioration of water quality, and the introduction of invasive species, primarily *Dreissena* spp. [7],[8],[9]. Negative effects of *Dreissena polymorpha* on unionids were first documented in Europe in the 1930s [10],[11]. By attaching to unionid valves, *Dreissena* hamper movement and burrowing, filter feeding, respiration, and reproduction [12],[13],[14], and induce shell deformities [15],[16],[17]. *Dreissena* may also directly compete with unionid bivalves for food [18],[19],[20],[21],[22], and occupy otherwise available space [23]. Heavy infestations of *Dreissena* may reduce glycogen reserves in host unionids [20],[24],[25] and cause depletion of their energy stores and total biomass [26]. While the causal mechanisms of unionid mortality as a result of dreissenid infestation are complex, the strong link between the level of infestation and mortality suggests that infestation intensity directly relates to the impact [26],[27], but see [22]. Thus, thresholds for lethal impact were predicted at 100 dreissenid per unionid, and/or mean dreissenid to unionid mass ratios ≥1.0 [26],[27]. Direct attachment was proven to be a very important component of the total effects of zebra mussel populations on unionids [28],[29].

In North America, sharp declines in diversity and abundance of native unionids after *Dreissena* invasion were best documented in lakes St. Clair and Erie, where both *Dreissena* species (*D. polymorpha*, the zebra mussel, and *D. rostriformis bugensis*, the quagga mussel) were first detected in the late 1980s – early 1990s [30],[31]. Historically, up to 40 species of unionids were recorded in Lake Erie, 27 in Lake St. Clair, and 39 species in the Detroit River [32],[33],[34]. A strong decline in diversity and abundance of unionids was already detected in lakes Erie and St. Clair before *Dreissena* invasion [35], and the arrival of dreissenids accelerated their decline [20],[36],[37]. The number of unionid species after the zebra mussel invasion in Lake St. Clair rapidly dropped from 18 prior to invasion in 1986 to 5 species in 1994 [38]. Thousands of attached *Dreissena* per unionid were reported in the early 1990s [39], and the mass of attached dreissenids was several times greater than the host unionid mass [37],[40], inducing high unionid mortality [27],[41].

The European experience suggested that the extensive infestation of unionids by *D. polymorpha*, resulting in mass mortality, is characteristic of periods of rapid zebra mussel population growth shortly after they invade a new waterbody, and the impact on unionids declines with time [13],[14],[42]. However, there is a lack of data from European waterbodies examining the effect of *D. r. bugensis* on unionids [42] due to the species’ recent introduction [43]. Although *D. polymorpha* and *D. r. bugensis* are closely related, share a native range, have a similar life history, and have both become important freshwater invaders throughout the northern hemisphere, they express several key differences [44],[45]. Quagga mussels with their lower rate of byssal thread production and attachment strength are less likely to resist dislodgment [46], and consequently their infestation and potential impacts on unionids may be less severe than those of zebra mussels. The lower Great Lakes, initially dominated by zebra mussels, have become dominated by quagga mussels since early 2000s [47],[48]. We do not know, however, how the presence of both dreissenid species may affect their impacts on unionids [45] and the impact of the ongoing replacement of zebra mussels with quagga mussels on unionids has not been investigated [42].

Susceptibility to *Dreissena* infestation may depend on the unionid species’ life history and ecological traits, such as burrowing activity, substrate preference, feeding behavior, brooding period, growth rate, or shell morphology [20],[49],[50],[51],[52],[53], but none of these characteristics have been examined on a large scale several decades after *Dreissena* invasion.

The purpose of the present study was to access the unionid infestation by *D. polymorpha* and *D. r. bugensis* in the nearshore waters of the lower Great Lakes region 25 years after the dreissenid invasion, addressing the following hypotheses: (1) dreissenid species differ in their intensity of unionid infestation, and (2) unionid species, depending on taxonomy or life history, differ in their resistance to dreissenid infestation. To address these hypotheses, in 2011–2012 we collected data on the level of dreissenid infestation present on unionids living in lakes Erie, Ontario, and St. Clair, the Detroit River, and three inland lakes in Michigan.

## Materials and Methods

### Study area and survey methods

**Figure 1:**
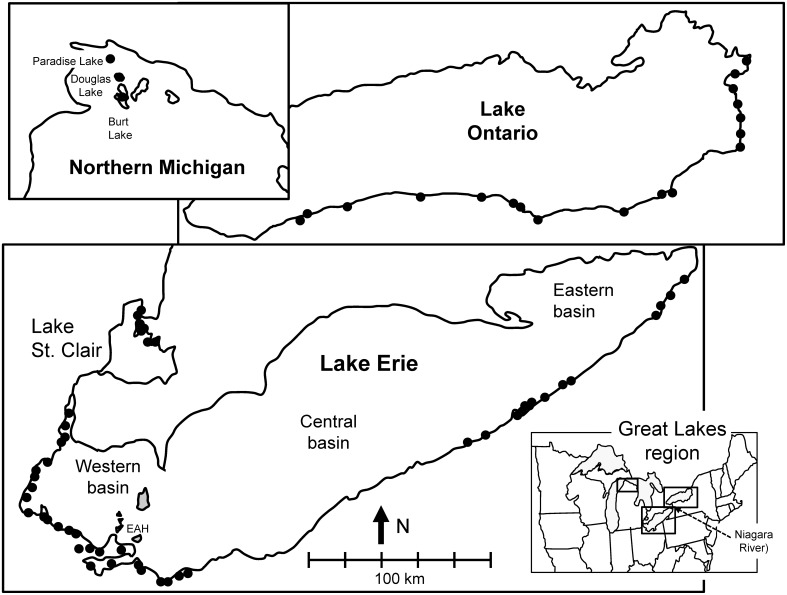
Map of sampling locations surveyed in 2011–2012. Each nearshore location (dots) was sampled at several (from 1 to 5) standard collection sites (0.5 ha surveyed for two person hours of search time). The inset gives sampling locations in inland Michigan lakes (Burt, Douglas, and Paradise).

Surveys focused on large areas of shallow waters hydrologically connected to the lake, with lentic flow and soft substrates, such as nearshore areas, mouths of tributary rivers, fringing wetlands, drowned river-mouth lakes, ponds, and marshes in the coastal zone of the lower Great Lakes (Fig. 1) [54]. Survey locations included areas where unionids were previously reported [54],[55],[56],[57],[58],[59],[60],[61]. At each location, unionids were sampled at several (from 1 to 5) sites. In 2011 and 2012 we surveyed 7 locations (17 sites) in Lake St. Clair, 4 locations (6 sites) in the Detroit River, 41 locations (114 sites) in Lake Erie [54], and 33 locations (54 sites) in Lake Ontario. Quagga mussels were the lake-wide dominant species in benthic dreissenid communities of lakes Erie and Ontario, while zebra mussels constituted 90% of *Dreissena* spp. in Lake St. Clair and the Detroit River [62],[63],[64] (Table 1). In addition, we collected data from waterbodies colonized by zebra mussel alone [63]: three inland lakes in Michigan in 2011 (three sites on Lake Paradise, Emmet County, three sites on Douglas Lake, Cheboygan County, and one site on Burt Lake, Cheboygan County).

**Table 1:** Infestation of unionids by *Dreissena* spp. in the waterbodies surveyed in 2011–2012.

Parameter	LakeErie	LakeOntario	LakeSt. Clair	BurtLake	DouglasLake	LakeParadise	Detroit River
Infested unionids	33.3%	29.1%	45.8%	97.8%	97.3%	90.8%	85.2%
(sample size)	(1,914)	(1,493)	(461)	(45)	(74)	(109)	(27)
*Dreissena* spp. to hostunionid wet weight ratios:							
mean ± standard error	0.065±0.01	0.061±0.007	0.64±0.05	0.65±0.08	1.09±0.18	0.55±0.08	n.r.
median (sample size)	0.005 (178)	0.019 (295)	0.38 (169)	0.670 (22)	0.718 (34)	0.350 (45)	n.r.
[Q25–Q75]	[0.0004–0.046]	[0.006–0.49]	[0.07–1.00]	[0.36–0.67]	[0.33–0.72]	[0.19–0.35]	
Unionids with past infestation	32% (1,580)	50% (1,301)	84% (461)	98% (45)	99% (74)	98% (109)	96% (27)
Uninfestedunionids of totalwith pastinfestation	47%	54%	47%	0%	1%	7%	12%
Time since invasion^a^ (years) by							
* D. polymorpha*	25	23	23	18	10	18	23
* D. r. bugensis*	22	21	22	-	-	-	22
* *reference	[31],[99]	[99],[100]	[30],[63]	[63]	[63]	[101]	[102]
*D. polymorpha* of total dreissenid abundance in the waterbody	5%	1%	90%	100%	100%	100%	90%
* *reference	[64]	[62]	b	[63]	[63]	[63]	b

Percent unionids infested by dreissenid mussels, the ratio of total wet weight of attached *Dreissena* spp. (>2 mm in size) to host unionid wet weight (mean ± standard error, median, lower and upper quartiles, sample size in parentheses), percent unionids with past infestation (unionids with or without *Dreissena* and with byssal threads) and percent *D. polymorpha* of total lake-wide dreissenid abundance are given for each waterbody studied (the lower Great Lakes, Lake St. Clair, the Detroit River, and inland lakes in north Michigan (Burt, Douglas, and Paradise lakes)).

a Time since the first recorded finding. No *D. r. bugensis* was reported from lakes Burt, Douglas and Paradise.

b D. Zanatta, unpublished data.

A standard collection site was an area of 100×50 m (0.5 ha). To standardize efforts across sites, two person hours of search time was spent per each 0.5 ha site, however, if the sampling location was small, half the standard area was searched for half the time. Sampling for unionids in each site was performed by hand collection by wading in shallow water, snorkel (up to 1.5 m depth), and SCUBA (1.5 to 3 m at only a few sites) applying tactile searches (probing fingers over and into sediment, usually up to 10 cm deep) at each site. Sampling times and person efforts to detect the presence of unionids and species were used to calculate catch-per-unit of effort data (mussels per person hour, unionids ph^−1^). Collected live unionids and shells were identified using published taxonomic guides [65],[66],[67],[68], measured to the nearest mm, and weighed. Live unionids were returned to the sediments from which they were taken.

Dreissenid infestation data were collected from sites where more than 5 individuals of any unionid species were obtained, a criterion met for 18 unionid species across lakes Erie and Ontario, and 16 species among Lake St. Clair, inland lakes in Michigan, and the Detroit River. To measure infestation we recorded the number of unionids with attached live *Dreissena* spp. and evidence of past infestation (i.e., presence of byssal threads on the shells with or without live dreissenids). Attached dreissenids were removed and total live wet weight of dreissenids per host unionid was recorded for each infested individual, but for no more than the first 10–15 specimens of each species. Weight of live unionid hosts (total live weight, g) was also recorded before returning them to the site area.

The dreissenids removed from unionids collected in the lakes where both dreissenid species were present were fixed in individual sample bags with ethanol and each sample was marked by location and site code and unique unionid number. All dreissenids collected were identified to species, individually weighed (±0.0001 g) and measured (length±0.01 mm) in the lab. To adjust for weight loss caused by preservation of samples in ethanol, we divided the measured weight by 0.77. This correction factor was determined by periodically weighing fixed zebra and quagga mussels (total weight, body plus shell) during 202 days to monitor the change in weight. We found a 23% (±1% SE, *n* = 29) average reduction in the original live weight of 10–20 mm mussels (the most prevalent group collected) that occurred between the 42th and 55th day since fixation, after which time mussel weight stabilized. This allowed us to re-calculate the initial weight of collected dreissenids that were all processed between 3 and 9 months after fixation.

### Data Analysis

Infestation levels were assessed as the proportion of unionids (1) with live dreissenids on their shells (prevalence of infestation), (2) with past infestation (with the presence of byssal threads on the shells) from the total number of unionids collected, and (3) the proportion of uninfected unionids (without attached live dreissenids but with byssal threads) of those with past infestation. We determined the number and total wet weight of each dreissenid species removed from each unionid host, and calculated the ratio of the *Dreissena* spp. wet weight from the host unionid weight (D/U). Finally, the proportion of *D. polymorpha* of (1) the combined number and (2) the combined wet weight of dreissenids (*D. polymorpha* and *D. r. bugensis*) per host unionid was calculated.

Data were tested for normality using a Shapiro-Wilk test. Parametric tests were used if original or transformed data met the normality assumption, otherwise, non-parametric tests were applied. As the distribution of dreissenids on host unionids was positively skewed (skewness = 4.16±0.08) and strongly peaked (kurtosis = 24.06±0.15), we used the median which is a better measure of central tendency than the mean in distributions strongly departing from normal [69]. Differences among waterbodies in the proportion of unionids infested were tested by a one-way ANOVA on logit-transformed [70] mean data for each location. To determine which species was more prevalent on unionid shells, we compared the number and weight of zebra *vs*. quagga mussels per host unionid using a paired *t*-test. We also tested if infestation parameters (the proportion of unionids infested, the proportion of unionids with past infestation, the D/U ratio, and the proportion of uninfested unionids of total with past infestation) were different for unionids collected in waterbodies dominated with either zebra or quagga mussels using two-sided *t*-tests on logit-transformed means calculated per each location. To test for change in the dreissenid/unionid weight ratio in waterbodies dominated by either zebra or quagga mussels, we compared current data with those collected from nearby sites in western Lake Erie in 1990 and the Detroit River in 1992 [41],[71]. The average ratio was calculated for each unionid species and then compared among three groups: (1) 1990–1993 data from western Lake Erie (at that time dominated by the zebra mussel) and the Detroit River, (2) 2011–2012 data from lakes Erie and Ontario dominated by the quagga mussel, and (3) 2011–2012 data from zebra mussel-dominated waterbodies, using a Friedman ANOVA.

We examined and compared the extent to which the level of infestation depended on the host unionid species *versus* the dominant *Dreissena* species in the waterbody by examining the proportion of unionids with dreissenid colonization, the proportion of unionids with past infestation, and the D/U ratio for unionid species found in both zebra and quagga mussel dominated waterbodies. We built linear models containing terms for the intercept, the dominant *Dreissena* species (2 levels), and the host unionid species, and examined their contribution to the model fit. To test if unionid species belonging to different tribes (Anodontini, Amblemini, Lampsilini, Pleurobemini, and Quadrulini) or life strategies (opportunistic, periodic, or equilibrium depending on their life span, maturity, growth rate and fecundity) [9] differed in their infestation by *Dreissena* in waterbodies dominated by zebra or quagga mussels, we applied a two-way Analysis of Similarities (ANOSIM). ANOSIM is a resampling technique that uses permutation/randomization methods on similarity matrices to identify differences among group of species [72] in PRIMER 6 software [73]. Matrices of measured parameters (proportion of infested unionids, unionids with past infestation, D/U ratios, and proportion of uninfested unionids with past infestation) were produced for each species where the number of mussels collected was >5 for waterbodies studied. These resemblance matrices were built using Euclidean distances on data that were log-transformed prior to analysis. For all tests, differences were considered statistically significant at *P*<0.05.

### Ethics statement

The work was carried out with appropriate collecting permits (Scientific License to Collect or Possess Freshwater Invertebrates issued by the New York State Department of Environmental Conservation; Scientific Collection Permit issued by Michigan Department of Natural Resources; Wild Animal Collecting Permit issued by Ohio Division of Wildlife, and Scientific Collector Permit issued by the Pennsylvania Fish and Boat Commission). Most of the research was done on public lands but if the land was private, landowner permission was acquired from each property owner before entering their property. No unionid species listed or candidates under U.S. Endangered Species Act were encountered during our study.

## Results

### Do dreissenid species differ in their intensity of unionid infestation?

A total of 4329 individual unionids of 26 species were collected from nearshore waters in the lower Great Lakes region. Fifteen species were found in each of the lakes Erie and St. Clair, 11 in Lake Ontario, 10 in the Detroit River, and from 2 to 5 species were found in three inland lakes of northern Michigan (S1 Table).

**Figure 2:**
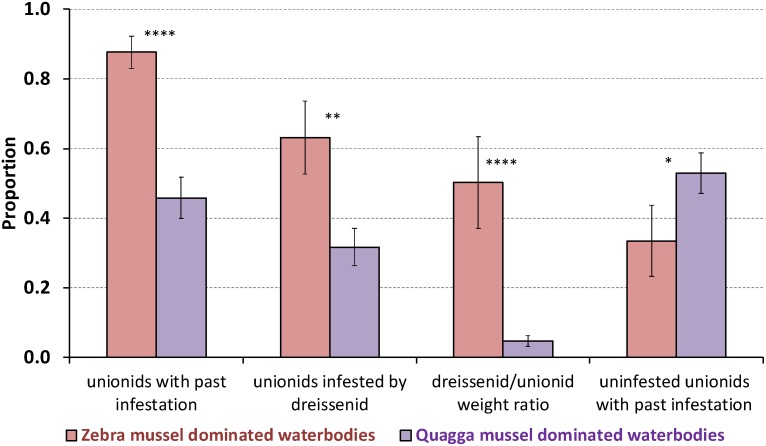
Infestation of unionids in waterbodies dominated by zebra or quagga mussels. Infestation parameters (averaged across sampled locations, mean ± standard error of the mean) include the proportion of unionids with past infestation (regardless of *Dreissena* presence), proportion of infested unionids, *Dreissena* spp. per host unionid wet weight ratio, and the proportion of uninfested unionids of those with past infestation in studied waterbodies (lakes Erie, Ontario, St. Clair, inland Michigan lakes (Paradise, Douglas and Burt), and the Detroit River) in 2011–2012. Lakes Erie and Ontario were predominantly occupied by quagga mussels (*Dreissena rostriformis bugensis*, purple bars) and all other waterbodies – by zebra mussels (*Dreissena polymorpha*, magenta bars). Infestation parameters of unionids collected from zebra- and quagga mussel-dominated lakes were significantly different (****P<0.0001; **P<0.01; *P<0.05, *t*-test).

The proportion of unionids infested by dreissenids varied significantly among waterbodies (*P* = 0.042, one-way ANOVA), and was lower in the nearshore areas of lakes Erie and Ontario colonized predominantly by quagga mussels compared to Lake St. Clair, the Detroit River, and inland lakes surveyed, which are colonized by zebra mussels (over 85%, Table 1). The differences between zebra mussel- and quagga mussel-dominated waterbodies were significant for all infestation parameters (proportion of infested unionids: *P* = 0.007; proportion of unionids with past infestation: *P* = 0.00007; D/U ratio: *P* = 0.00001; and proportion of uninfested unionids with evidence of past infestation: *P* = 0.047, *t*-tests, Fig. 2).

Only a third of all unionids collected in lakes dominated by quagga mussels (lakes Erie and Ontario) were infested by dreissenids (Table 1, S1 Table). Between 30 and 50% of all unionids (with and without attached dreissenids) collected in these lakes showed evidence of past infestation (remains of byssal threads attached to unionid valves). Furthermore, almost half of all unionids with past infestation had no attached dreissenids on their shells. The median number of attached *Dreissena* was 3 per unionid (Table 2); a third of all infested unionids had <2 dreissenids attached to their shell, and 70% had <6 dreissenids. The average ratio of the mass of attached *Dreissena* to that of the host unionid (D/U ratio) was <10% (Table 1).

**Table 2:** Numbers and wet weight of all attached dreissenids (>2 mm in size) and separately by species (*Dreissena polymorpha* and *D. rostriformis bugensis*) per infested host unionid (mean ± standard error, median, lower and upper quartiles, sample size) in studied lakes in 2011–2012.

Parameter	Lake St. Clair	Lake Erie	Lake Ontario
Number attached dreissenids:			
mean ± standard error (sample size)	12.6±1.1 (133)	10.0±0.9 (499)	7.9±0.9 (372)
range	1–56	1–196	1–151
median (Q25–Q75)	8 (2–20)	3 (1–8)	2 (1–5)
Number *D. polymorpha* per host unionid:			
mean ± standard error (sample size)	8.8±0.8 (133)	7.4±0.56 (499)	4.5±0.5 (298)
median (Q25–Q75)	6 (1–13)	3 (1–7)	1 (1–4)
Number *D. r. bugensis* per host unionid:			
mean ± standard error (sample size)	3.8±0.5 (133)	2.6±0.5 (499)	3.4±0.7 (299)
median (Q25–Q75)	2 (1–6)	0	0
Wet weight of *D. polymorpha*per host unionid (g):			
mean ± standard error (sample size)	7.65±0.69 (133)	2.21±0.33 (499)	3.68±0.40 (298)
median (Q25–Q75)	5.0 (1–10.2)	0.5 (0.2–1.4)	1.0 (0.4–3.0)
Wet weight of *D. r. bugensis*per host unionid (g):			
mean ± standard error (sample size)	2.44±0.31 (133)	0.35±0.13 (499)	1.34±0.29 (296)
median (Q25–Q75)	0.9 (0–3.3)	0	0 (0–0.2)
Proportion *D. polymorpha* wetweight of total dreissenid weight(per host unionid):			
mean ± standard error (sample size)	0.80±0.02 (133)	0.93±0.01 (499)	0.87±0.02 (298)
median (Q25–Q75)	0.9 (0.7–1.0)	1.0 (1.0–1.0)	1.0 (0.9–1.0)

In contrast, the proportion of unionids infested by *Dreissena* was much higher in waterbodies dominated by zebra mussels (46% in Lake St. Clair, 85% in the Detroit River, and >90% in inland Michigan lakes). Likewise, the proportion of unionids with past infestation (>80% for all zebra mussel-dominated lakes) was higher than in lakes Erie and Ontario (Table 1). The average D/U ratio in zebra mussel dominated waterbodies was an order of magnitude higher than in quagga mussel-dominated lakes, and was nearly 1 (i.e., dreissenid weight almost equal to the host unionid weight) (Table 1).

D/U ratio by unionid species differed significantly not only among samples collected from lakes dominated by either quagga or zebra mussels in 2011–2012, but also among samples collected in western Lake Erie in 1990–1993 when it was dominated by zebra mussels (*P* = 0.028, Friedman ANOVA, Table 3).

**Table 3:** Average ratio *Dreissena* spp. weight: host unionid weight for unionid species collected in waterbodies dominated by different *Dreissena* species.

Unionid species	*D. polymorpha*-dominated western Lake Erie (1990) and the Detroit River (1992)^a^	*D. polymorpha*-dominated lakes (St. Clair, Burt, Douglas, Paradise) (2011–2012)^b^	*D. r. bugensis*-dominated lower Great Lakes (2011–2012)^b^
*Elliptio* spp.	0.674	0.416	0.072
*Lampsilis* spp.	0.734	0.672	0.083
*Lasmigona costata*	0.264	0.324	
*Leptodea fragilis*	1.357	0.003	0.102
*Ligumia* spp.	0.510	0.915	0.005
*Potamilus alatus*	0.377	0.493	0.126
*Villosa iris*	0.953	0.877	
*Fusconaia flava*	0.989	1.223	
*Amblema plicata*	0.537	0.017	0.080
*Pyganodon grandis*	1.936	0.119	0.039
*Quadrula quadrula*	0.424		0.026

Unionids were collected during the peak of *Dreissena polymorpha* invasion in western Lake Erie (1990) and in the Detroit River (1992) [71], and in present study from *D. polymorpha*-dominated lakes (St. Clair, Burt, Douglas, Paradise) and *D. r. bugensis*-dominated lakes (Erie, Ontario).

a total dry weight was used.

b total wet weight was used.

Across waterbodies where both dreissenid species were present, most of the infested unionids (90%) had more zebra than quagga mussels attached to their shells. The mean number of zebra mussels per host unionid was approximately twice that of quagga mussels (*P*<<0.001, paired *t*-test) across all sampled sites (Table 2). Likewise, the average weight of attached zebra mussels per unionid was also significantly higher than that of quagga mussels (*P*<<0.001, paired *t*-test). Zebra mussel weight comprised over 80% of the total dreissenid biomass per host unionid (Table 2).

### Do unionid species, depending on taxonomy or life history, differ in their resistance to dreissenid infestation?

We found no significant differences among tribes of Unionidae in their infestation by *Dreissena* across all lakes (Global *R* = −0.075, *P* = 0.66), but there was a significant effect of dominant *Dreissena* species (Global *R* = 0.644, *P* = 0.002, two-way ANOSIM). Similarly, there was no difference in dreissenid infestation among unionid species with different life strategies (Global *R* = −0.037, *P* = 0.57), but infestation parameters were significantly different between zebra mussel- and quagga mussel-dominated lakes (Global *R* = 0.609, *P* = 0.001, two-way ANOSIM).

**Figure 3:**
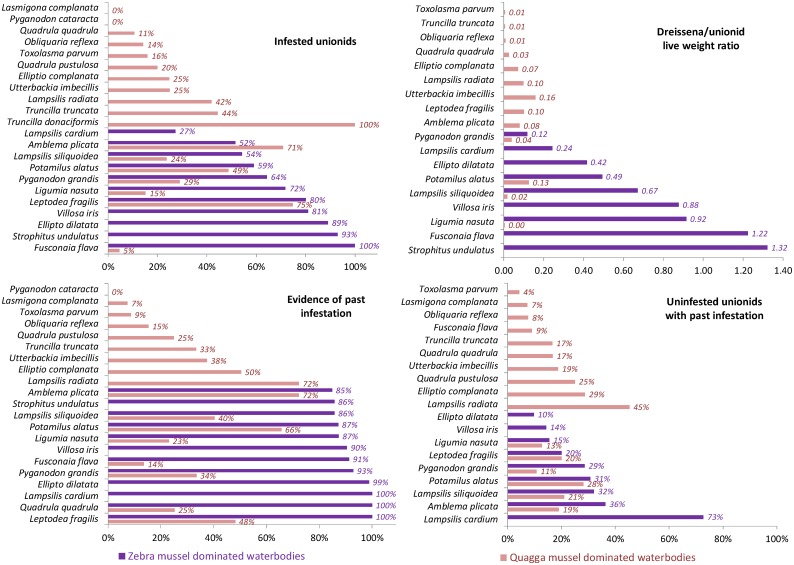
Infestation parameters of unionids by species collected from waterbodies dominated by zebra or quagga mussels. Proportion of unionids infested by dreissenids by unionid species, proportion of unionids with evidence of past infestation (regardless of *Dreissena* presence), *Dreissena* spp./host unionid wet weight ratios, and the proportion of uninfested unionids of those with past infestation in waterbodies dominated by *D. polymorpha* (Lake St. Clair, the Detroit River, Paradise, Douglas and Burt lakes in Michigan, magenta bars) and in lakes Erie and Ontario dominated by *D. r. bugensis* (purple bars).

Although the infestation parameters (the proportion of infested unionids, the proportion of unionids with past infestation, and the D/U ratios) varied to some extent among unionid species (Fig. 3), the effect of the dominant *Dreissena* species had a much stronger impact on infestation for unionid species present in both zebra and quagga mussel dominated waterbodies (linear regression analysis on proportion infested: 8 species, unionid species effect: −0.014, *P* = 0.661, *Dreissena* effect: 0.305, *P* = 0.0339; past infestation: 8 species, unionid species effect 0.01, *P* = 0.553, *Dreissena* effect: 0.47, *P* = 0.0002; D/U ratio: 6 species, unionid species effect: −0.12, *P* = 0.131, *Dreissena* effect: 0.50, *P* = 0.018). Therefore the effects of dreissenid species were much more important than species-specific differences in unionids.

## Discussion

We found that occurrence and intensity of unionid infestation by *Dreissena* spp. have declined since initial invasion, and the number of dreissenids attached to unionids in the lower Great Lakes has decreased to almost one tenth of the numbers found in the early 1990s. Zebra mussels were found to infest unionids much more frequently and in greater numbers than quagga mussels. Consequently, the proportion of infested unionids, and the number and weight of attached dreissenids were all lower in waterbodies where the quagga mussel was more numerous. We found no evidence, however, to suggest a difference in *Dreissena* infestation among unionid species of different tribes or life strategies.

### Dreissenid species do differ in their intensity of unionid infestation

In many waterbodies where both zebra and quagga mussels co-occur, quagga mussels eventually outcompete zebra mussels [44],[45]. A number of hypotheses have been suggested to explain the displacement of zebra mussels by quagga mussels, including the earlier time of quagga mussel reproduction that could affect recruitment and population abundance; higher feeding and bioenergetic efficiency of quagga mussels, resulting in positive growth over a wider range of food concentrations compared to zebra mussels which lose weight under low food levels (reviewed in [45]). Currently, in the lower Great Lakes zebra mussels remain common only in the shallow western basin of Lake Erie, where they compose 33% of the combined dreissenid density, in contrast to the central and eastern basins (∼2%) [64]. In Lake Ontario, the relative abundance of zebra mussels in the shallow areas declined from 63% in 1995 to 1% by 2003 [48].

The ongoing replacement of zebra mussels by quagga mussels in the lower Great Lakes is likely the most important driver for the current lower unionid infestation rates compared to the 1990s. Current levels of unionid infestation with *Dreissena* are much lower than in the early 1990s when an average of >200 dreissenids/unionid was reported from Lake Erie [20],[36]. In 2011–2012, only one third of all unionids collected in lakes Erie and Ontario were infested, although half of unionids without *Dreissena* had evidence of past infestation.

In contrast, in waterbodies predominantly colonized by zebra mussels (Michigan lakes, the Detroit River and Lake St. Clair), unionid infestation was almost three times higher than in quagga mussel-dominated lakes (Erie and Ontario), despite that these waterbodies were colonized by dreissenids at approximately the same time. The mean mass of attached dreissenids in zebra mussel-dominated lakes was an order of magnitude higher that in quagga mussel-dominated lakes, and was almost equal to the host unionid mass, a quantity near the predicted threshold for lethal impacts [26]. The effect of the dominant dreissenid species on the level of unionid infestation was significant not only across systems (i.e. in different lakes dominated by different dreissenid species) but also temporally in the same waterbodies (e.g. Lake Erie dominated by zebra mussels in early 1990s *vs*. in 2011–2012 when dominated by quagga mussels, Table 3). We note that the overall dreissenid densities in these waterbodies have likely declined over the past decade [64],[74]. Previous studies from waterbodies in Europe and North America indicate a tendency for dreissenid impacts on unionids to decline with time [13],[14],[21],[25],[42],[75]. However, our results from waterbodies colonized by dreissenids for over two decades strongly suggest that the effect of dreissenid species replacement on the level of unionid infestation exceeds the effect of time since dreissenid invasion.

Why were zebra mussels more abundant on unionid shells in both lakes Erie and Ontario where they have been almost completely replaced by quagga mussels in the benthic community? Zebra mussel “refugia” in Great Lakes include nutrient-rich bays and tributaries where unionids are found [76], as well as upper littoral zones of the lakes [77] where they grow faster at higher food concentrations [78] than quagga mussels. Zebra mussels are more likely to resist dislodgment due to their flattened ventral shell surface and higher rate of byssal thread production [46],[79],[80], and thus are likely to be better adapted to the unstable environments where fluctuations in water currents, temperature, and waves are prominent [44],[45]. Therefore, their relative proportion to quagga mussels on substrates like buoys, floating docks, macrophytes, and boats is much higher than on the lake bottom [77], similarly to what we found on unionid shells. The limited information available from other waterbodies where both dreissenids are present also suggests that zebra mussels are more commonly found on unionid shells [27],[76],[81],[82]. The survival of quagga mussels attached to unionid shells may also be lower due to higher predation pressure compared to zebra mussels [59],[83] as their thinner shells are more easily crushed and digested by fish [84],[85]. Lower survivorship of quagga mussels after aerial exposures [86] and a lower upper temperature tolerance compared to the zebra mussel [87] may likewise be critical in shallow waters, especially where water levels fluctuate [76]. Finally, more complex interactions may contribute to differences in establishment patterns and survival between these dreissenid species, including substrate preferences, colonization and post-settling survivorship [85], and potential competition for food resources [78] in shallow and nutrient-rich nearshore wetland areas [76].

One approach to evaluate or predict community or ecosystem impacts of species, especially invaders, uses functional groups or functional traits [88],[89],[90]. Functional traits, linking environmental conditions to species performance, provide a basis for understanding how the traits of individuals scale up to determine community structure and function, and for developing predictive models of ecosystem functioning that are based on physiologically relevant organism properties [88],[89]. The relationship between morphology and functional performance, however, can often be nonlinear [91]. Guild and functional approaches, useful for identification of potential pests or their mitigation, may be less applicable for qualifying consequences of invasion that are linked to specific species characteristics [92]. Both *D. polymorpha* and *D. r. bugensis* are often considered to be similar in terms of the impacts they may have on communities or ecosystems [93],[94],[95]. However, while these two species are taxonomically closely related and belong to the same functional group, they are not identical. We suggest that small differences between these congeners in a context-dependent performance may have large consequences for the communities they invade.

### Do unionid species, depending on taxonomy or life history, differ in their resistance to dreissenid infestation?

Some differences in mortality among unionid species infested by *Dreissena* have been attributed to differences in life history and ecological traits (like burrowing activity, substrate preference, feeding behavior, brooding period, growth rate) or shell morphology [20],[49], [50],[51],[52],[53]. However, conflicting results were found among species: for example, heavy shelled species (e.g., Amblemini, Pleurobemini, and Quadrulini) were suggested to be more tolerant to dreissenid fouling than light shelled Anodontinae [20], but the unionids “returning” to western Lake Erie are more often thin-shelled, fast growing species (e.g., *L. fragilis* and *P. grandis*) [96]. In different studies, long-term brooders (e.g., Anodontinae and Lampsilini) were found to be more susceptible to *Dreissena* impact than short-term brooders (e.g., Amblemini, Pleurobemini, and Quadrulini) [50],[71],[97], or less susceptible than short-term brooders [16]. Our analyses, however, indicated that taxonomic grouping was not a significant factor related to infestation by *Dreissena*.

Likewise, we found no significant differences in infestation rates for species based on their life history strategies. Unionid life history strategies can potentially affect their sensitivity to disturbance [9]: species with opportunistic life strategy (e.g., Anodontini and Lampsilini with short life span, early maturity, high fecundity and growth rate) are good colonizers and can persist in disturbed and unstable but productive habitats. In contrast, species with equilibrium strategy (e.g., Amblemini, Quadrulini with long life span and late maturity, and low fecundity) are hypothesized to favor stable productive habitats, and periodic strategists (e.g., Alasmidonta spp.) are in intermediate position [9]. We found that dreissenid infestation – one of the main contemporary disturbances faced by native mussels – differed little among unionid species while the most important predictor of infestation level was the dominant dreissenid species in the lake. It should be emphasized, however, that we tested these hypotheses on the species that have largely survived the *Dreissena* invasion by being adapted to the specific habitats of coastal nearshore areas we surveyed.

## Conclusions

Determining where and when impacts of invasive species will be most detrimental is critical for the development of global management priorities both after and in advance of future invasions, for example of other exotic epifaunal byssate bivalves such as *Limnoperna fortunei* [98]. When multiple species invade, we also need to know what kind of interactions - additive, superadditive (“invasional meltdown”), or subadditive (“invasional interference”) will occur for both predicting impacts of invasive species and for prioritization of management efforts [4]. This is the first large-scale systematic study revealing the differences between two most notorious aquatic invaders - quagga and zebra mussel – in their rate of infestation. The decrease in unionid infestation that accompanied the competitive replacement of zebra with quagga mussels may result in reduced overall impact on unionid community. However, as infestation accounts for a large part, but not all of the impact [22], the magnitude of such effects remains to be evaluated. For management purposes, our priorities should be in preventing the spread of dreissenids, but if a waterbody is already invaded by zebra mussels, it seems unlikely that the subsequent invasion of quagga mussels will greatly increase the net impact on unionid community. Based on our data, we predict a lower unionid infestation, and thus perhaps lesser impact when the quagga mussel is the sole dreissenid invader; however, more research in lakes invaded only by quagga mussels are needed to validate that hypothesis.

Functional groups or functional traits are commonly used to evaluate or predict community or ecosystems impacts of species, and often taxonomically and functionally similar species, including important groups of invaders, are expected to have similar community or ecosystem impacts. This seems justifiable for *Dreissena* spp. as both share a native habitat, a common morphology, life history, and have both become important invaders of freshwaters throughout the northern hemisphere. However, we found that some differences (e.g., different rate of byssal thread production and attachment strength) between these two dreissenid species may have large consequences for native communities they invade. Now, even two decades after invasion, we find a stronger infestation of unionids in waterbodies dominated by the zebra mussel compared to those dominated by the quagga mussel. Therefore, researchers should be cautious while using functional groups as the unit of study both in conservation efforts and in predicting community or ecosystem impacts of invasive species, as small dissimilarities between even closely related species can drive profound differences in environmental impacts and affect restoration or conservation efforts.

## Supporting Information

S1 Table **Unionid species collected in waterbodies surveyed in 2011–2012 and their average infestation by** ***Dreissena*** **spp.**
(XLSX)

## References

[1] Simberloff D, Von Holle B (1999) Positive interactions of nonindigenous species: invasional meltdown? Biol Invasions 1:21–32.

[2] Yang SA, Ferrari MJ, Shea K (2011) Pollinator behavior mediates negative interactions between two congeneric invasive plant species. Am Nat 177:110–118. 21117967 10.1086/657433

[3] Simberloff D (2006) Invasional meltdown 6 years later: important phenomenon, unfortunate metaphor, or both? Ecol Lett 9:912–919. 16913932 10.1111/j.1461-0248.2006.00939.x

[4] Rauschert ESJ, Shea K (2012) Invasional interference due to similar inter- and intraspecific competition between invaders may affect management. Ecol Appl 22:1413–1420. 22908701 10.1890/11-2107.1

[5] Lydeard C, Clark SA, Perez KE, Cowie RH, Ponder WF, et al (2004) The global decline of nonmarine mollusks. Bioscience 54:321–330.

[6] Bogan AE (2008) Global diversity of freshwater mussels (Mollusca, Bivalvia) in freshwater. Hydrobiologia 595:139–147.

[7] Bogan AE (1993) Freshwater bivalve extinctions (Molusca: Unionidae): a search for causes. Am Zool 33:599–609.

[8] Strayer DL, Downing JA, Haag WR, King TL, Layzer JB, et al (2004) Changing perspectives on pearly mussels, North America’s most imperiled animals. Bioscience 54:429–439.

[9] Haag WR (2012) North American Freshwater Mussels: Natural History, Ecology, and Conservation. New York: Cambridge University Press. 505 p.

[10] Sebestyén O (1937) Colonization of two new fauna-elements of Pontus-origin (*Dreissena polymorpha* Pall. and *Corophium curvispinum* G. O. Sars *forma devium* Wundsch) in Lake Balaton. Verh Internat Verein Limnol 8:169–181.

[11] Dussart GBJ (1966) Limnologie. Paris: Gauthier-Villars. 678 p.

[12] Strayer DL (1999) Effects of alien species on freshwater mollusks in North America. J N Am Benthol Soc 18:74–98.

[13] Karatayev AY, Burlakova LE, Padilla DK (1997) The effects of *Dreissena polymorpha* (Pallas) invasion on aquatic communities in eastern Europe. J Shellfish Res 16:187–203.

[14] Burlakova LE, Karatayev AY, Padilla DK (2000) The impact of *Dreissena polymorpha* (Pallas) invasion on unionid bivalves. Int Rev Hydrobiol 85:529–541.

[15] Lewandowski K (1976) Unionidae as a substratum for *Dreissena polymorpha* . Pol Arch Hydrobiol 23:409–420.

[16] Hunter RD, Bailey JF (1992) *Dreissena polymorpha* (zebra mussel): Colonization of soft substrata and some effects on unionid bivalves. Nautilus 106:60–67.

[17] Schloesser DW, Nalepa TF, Mackie GL (1996) Zebra mussel infestation of unionid bivalves (Unionidae) in North America. Am Zool 36:300–310.

[18] Mackie GL (1991) Biology of the exotic zebra mussel, *Dreissena polymorpha*, in relation to native bivalves and its potential impact in Lake St. Clair. Hydrobiologia 219:251–268.

[19] Hebert PDN, Wilson CC, Murdoch MH, Lazar R (1991) Demography and ecological impacts of the invading mollusk *Dreissena polymorpha* . Can J Zoo 69:405–409.

[20] Haag WR, Berg DJ, Garton DW, Farris JL (1993) Reduced survival and fitness in native bivalves in response to fouling by the introduced zebra mussel (*Dreissena polymorpha*) in western Lake Erie. Can J Fish Aquat Sci 50:13–19.

[21] Strayer DL, Malcom HM (2007) Effects of zebra mussels (*Dreissena polymorpha*) on native bivalves: the beginning of the end or the end of the beginning? J N Am Benthol Soc 26:111–122.

[22] Strayer DL, Malcom HM (2014) Long-term change in the Hudson River’s bivalve populations: A history of multiple invasions (and recovery?). In: Nalepa TF, Schloesser DWeditors. Quagga and Zebra Mussels: Biology, Impacts, and Control. Boca Raton, FL: CRC Press. pp. 71–82.

[23] Tucker JK (1994) Colonization of unionid bivalves by the zebra mussel, *Dreissena polymorpha*, in pool 26 of the Mississippi River. J Freshw Ecol 9:129–134.

[24] Baker SM, Hornbach DJ (1997) Acute physiological effects of zebra mussel (*Dreissena polymorpha*) infestation on two unionid mussels, *Actinonaias ligamentina* and *Amblema plicata* . Can J Fish Aquat Sci 54:512–519.

[25] Bódis E, Tóth B, Sousa R (2014) Impact of *Dreissena* fouling on the physiological condition of native and invasive bivalves: interspecific and temporal variations. Biol Invasions 16:1373–1386.

[26] Ricciardi A, Whoriskey FG, Rasmussen JB (1995) Predicting the intensity and impact of *Dreissena* infestation on native unionid bivalves from *Dreissena* field density. Can J Fish Aquat Sci 52:1449–1461.

[27] Ricciardi A, Whoriskey FG, Rasmussen JB (1996) Impact of the *Dreissena* invasion on native unionid bivalves in the upper St. Lawrence River. Can J Fish Aquat Sci 53:1434–1444.

[28] Baker SM, Hornbach DJ (2000) Physiological status and biochemical composition of a natural population of unionid mussels (*Amblema plicata*) infested by zebra mussels (*Dreissena polymorpha*). Am Midl Nat 143:443–452.

[29] Baker SM, Hornbach DJ (2008) Zebra mussels (*Dreissena polymorpha*) attached to native mussels (Unionidae) or inanimate substrates: comparison of physiological rates and biochemical composition. Am Midl Nat 160:20–28.

[30] Hebert PDN, Muncaster BW, Mackie GL (1989) Ecological and genetic studies on *Dreissena polymorpha* (Pallas): a new mollusc in the Great Lakes. Can J Fish Aquat Sci 46:1587–1591.

[31] Carlton JT (2008) The zebra mussel *Dreissena polymorpha* found in North America in 1986 and 1987. J Great Lakes Res 34:770–773.

[32] Goodrich C, van der Schalie HI (1932) The naiad species of the Great Lakes. Occas Pap Mus Zool Univ Mich 238:8–14.

[33] Metcalfe-Smith JL, Staton SK, Mackie GL, Lane NM (1998) Changes in the biodiversity of freshwater mussels in the Canadian waters of the lower Great Lakes drainage basin over the past 140 years. J Great Lakes Res 24:845–858.

[34] Graf DL (2002) Historical biogeography and late glacial origin of the freshwater pearly mussel (Bivalvia: Unionidae) faunas of Lake Erie, North America. Occas Pap Mollusks 6:175–211.

[35] Nalepa TF, Manny BA, Roth JC, Mozley SC, Schloesser DW (1991) Long-term decline in freshwater mussels (Bivalvia: Unionidae) of the western basin of Lake Erie. J Great Lakes Res 17:214–219.

[36] Gillis PL, Mackie GL (1994) Impact of the zebra mussel, *Dreissena polymorpha*, on populations of Unionidae (Bivalvia) in Lake St. Clair. Can J Zool 72:1260–1271.

[37] Schloesser DW, Nalepa TF (1994) Dramatic decline of unionid bivalves in offshore waters of western Lake Erie after infestation by the zebra mussel, *Dreissena polymorpha* . Can J Fish Aquat Sci 51:2234–2242.

[38] Nalepa TF, Hartson DJ, Gostenik GW, Fanslow DL, Lang GA (1996) Changes in the freshwater mussel community of Lake St. Clair: from Unionidae to *Dreissena polymorpha* in eight years. J Great Lakes Res 22:354–369.

[39] Schloesser DW, Kovalak WP (1991) Infestation of unionids by *Dreissena polymorpha* in a power plant canal in Lake Erie. J Shellfish Res 10:355–359.

[40] Schloesser DW, Masteller EC (1999) Mortality of unionid bivalves (Mollusca) associated with dreissenid mussels (*Dreissena polymorpha* and *D. bugensis*) in Presque Isle Bay, Lake Erie. Northeast Nat 6:341–352.

[41] Schloesser DW, Kovalak WP, Longton GD, Ohnesorg KL, Smithee RD (1998) Impact of zebra and quagga mussels (*Dreissena* spp.) on freshwater unionids (Bivalvia: Unionidae) in the Detroit River of the Great Lakes. Am Midl Nat 140:299–313.

[42] Lucy FE, Burlakova LE, Karatayev AY, Mastitsky SE, Zanatta DT (2014) Zebra mussel impacts on unionids: a synthesis of trends in North America and Europe. In: Nalepa TF, Schloesser DWeditors. Quagga and Zebra Mussels: Biology, Impacts, and Control. Boca Raton, FL: CRC Press. pp. 623–646.

[43] bij de Vaate A, van der Velde G, Leuven RSEW, Heiler KCM (2014) Spread of the quagga mussel (Dreissena rostriformis bugensis) in Western Europe. In: Nalepa TF, Schloesser DWeditors. Quagga and Zebra Mussels: Biology, Impacts, and Control. Boca Raton, FL: CRC Press. pp. 83–92.

[44] Karatayev AY, Burlakova LE, Mastitsky SE, Padilla DK, Mills EL (2011) Contrasting rates of spread of two congeners, *Dreissena polymorpha* and *Dreissena rostriformis bugensis*, at different spatial scales. J Shellfish Res 30:923–931.

[45] Karatayev AY, Burlakova LE, Padilla DK (2014) General overview of zebra and quagga mussels: what we do and do not know. In: Nalepa TF, Schloesser DWeditors. Quagga and Zebra Mussels: Biology, Impacts, and Control. Boca Raton, FL: CRC Press. pp. 695–703.

[46] Peyer SM, McCarthy AJ, Lee CE (2009) Zebra mussels anchor byssal threads faster and tighter than quagga mussels in flow. J Exp Biol 212:2026–2035. 10.1242/jeb.028688 19525429

[47] Patterson MWR, Ciborowski JJH, Barton DR (2005) The distribution and abundance of *Dreissena* species (Dreissenidae) in Lake Erie, 2002. J Great Lakes Res 31 Suppl 2223–237.

[48] Watkins JM, Dermott R, Lozano SJ, Mills EL, Rudstam LG, et al (2007) Evidence for remote effects of dreissenid mussels on the amphipod *Diporeia*: analysis of Lake Ontario benthic surveys, 1972–2003. J Great Lakes Res 33:642–657.

[49] Arter HE (1989) Effect of eutrophication on species composition and growth of freshwater mussels (Mollusca, Unionidae) in Lake Hallwil (Aargau, Switzerland). Aquat Sci 51:87–99.

[50] Nalepa TF (1994) Decline of native unionid bivalves in Lake St. Clair after infestation by the zebra mussel, *Dreissena polymorpha* . Can J Fish Aquat Sci 51:2227–2233.

[51] Nichols SJ, Wilcox DA (1997) Burrowing saves Lake Erie clams. Nature 389:921–921.

[52] Allen DC, Vaughn CC (2009) Burrowing behavior of freshwater mussels in experimentally manipulated communities. J N Am Benthol Soc 28:93–100.

[53] Sousa R, Pilotto F, Aldridge DC (2011) Fouling of European freshwater bivalves (Unionidae) by the invasive zebra mussel (*Dreissena polymorpha*). Freshw Biol 56:867–876.

[54] Zanatta DT, Bossenbroek JM, Burlakova LE, Crail T, de Szalay F, et al. Distribution of native mussel (Unionidae) assemblages in coastal Lake Erie, Lake St. Clair, and connecting channels, twenty-five years after the dreissenid invasion. Northeast Nat In press.

[55] Nichols SJ, Wilcox D (1997) Coexistence of zebra mussels and native clams in a lake coastal wetland. J Shellfish Res 16:346.

[56] Nichols SJ, Amberg J (1999) Co-existence of zebra mussels and freshwater unionids: population dynamics of *Leptodea fragilis* in a coastal wetland infested with zebra mussels. Can J Zool 77:423–432.

[57] Zanatta DT, Mackie GL, Metcalfe-Smith JL, Woolnough DA (2002) A refuge for native freshwater mussels (Bivalvia: Unionidae) from impacts of the exotic zebra mussel (*Dreissena polymorpha*) in Lake St. Clair. J Great Lakes Res 28:479–489.

[58] Bowers R, de Szalay FA (2004) Effects of hydrology on unionids (Unionidae) and zebra mussels (Dreissenidae) in a Lake Erie coastal wetland. Am Midl Nat 151:286–300.

[59] Bowers R, Sudomir JC, Kershner MW, de Szalay FA (2005) The effects of predation and unionid burrowing on bivalve communities in a Laurentian Great Lake coastal wetland. Hydrobiologia 545:93–102.

[60] Bowers R, de Szalay FA (2007) Fish predation of zebra mussels attached to *Quadrula quadrula* (Bivalvia: Unionidae) and benthic molluscs in a Great Lakes coastal wetland. Wetlands 27:203–208.

[61] McGoldrick DJ, Metcalfe-Smith JL, Arts MT, Schloesser DW, Newton TJ, et al (2009) Characteristics of a refuge for native freshwater mussels (Bivalvia: Unionidae) in Lake St. Clair. J Great Lakes Res 35:137–146.

[62] Pennuto C, Howell ET, Lewis TK, Makarewicz JC (2012) *Dreissena* population status in nearshore Lake Ontario. J Great Lakes Res 38:161–170.

[63] Fuller P (2014) Nonindigenous Aquatic Species Database http://nas.er.usgs.gov.U.S. Geological Survey, Gainesville, FL. Accessed 14 May 2014.

[64] Karatayev AY, Burlakova LE, Pennuto C, Ciborowski J, Karatayev VA, et al (2014) Twenty five years of changes in *Dreissena* spp. populations in Lake Erie. J Great Lakes Res 40:550–559.

[65] Cummings KS, Mayer CA (1992) Field guide to freshwater mussels of the Midwest. INHS Manual 5: 194 p.

[66] Strayer DL, Jirka KJ (1997) The Pearly Mussels of New York State. NY State Mus Mem 26: 133pp. +127 plates.

[67] Spoo A (2008) The Pearly Mussels of Pennsylvania. Landisville, PA: Coachwhip Publications. 212 p.

[68] Watters GT, Hoggarth MA, Stansbery DH (2009) The Freshwater Mussels of Ohio. Columbus, OH: The Ohio State University Press. 421 p.

[69] Zar HJ (2010) Biostatistical Analysis. Upper Saddle River, NJ: Pearson Prentice-Hall. 944 p.

[70] Warton DI, Hui FKC (2011) The arcsine is asinine: the analysis of proportions in ecology. Ecology 92:3–10. 21560670 10.1890/10-0340.1

[71] Schloesser DW, Metcalfe-Smith JL, Kovalak WP, Longton GD, Smithee RD (2006) Extirpation of freshwater mussels (Bivalvia: Unionidae) following the invasion of dreissenid mussels in an interconnecting river of the Laurentian Great Lakes. Am Midl Nat 155:307–320.

[72] Clarke KR (1993) Non-parametric multivariate analyses of changes in community structure. Aust J Ecol 18:117–143.

[73] Clarke KR, Gorley RN (2006) PRIMER v6: User manual/tutorial. Plymouth, UK: PRIMER-E. 192 p.

[74] Lozano SJ (2011) Status of Macroinvertebrates in Lake Ontario. Final Report. Interagency Agreement US EPA-DOC/NOAA. Ann Arbor, MI: DOC/NOAA, Great Lakes Environmental Laboratory. pp. 1–33.

[75] Karatayev AY, Burlakova LE, Padilla DK (2002) Impacts of zebra mussels on aquatic communities and their role as ecosystem engineers. In: Leppakoski E, Gollach S, Olenin Seditors. Invasive Aquatic Species of Europe: Distribution, Impacts and Management. Dordreicht, The Netherlands: Kluwer Academic Publishers. pp. 433–446.

[76] Sherman JJ, Murray BA, Woolnough DA, Zanatta DT, Uzarski DG (2013) Assessment of remnant unionid assemblages in a selection of Great Lakes coastal wetlands. J Great Lakes Res 39:201–210.

[77] Karatayev VA, Karatayev AY, Burlakova LE, Padilla DK (2013) Lakewide dominance does not predict the invader species for dreissenids. J Great Lakes Res 39:622–629.

[78] Baldwin BS, Mayer MS, Dayton J, Pau N, Mendilla J, et al (2002) Comparative growth and feeding in zebra and quagga mussels (*Dreissena polymorpha* and *Dreissena bugensis*): implications for North American lakes. Can J Fish Aquat Sci 59:680–694.

[79] Claxton WT, Wilson AB, Mackie GL, Boulding EG (1998) A genetic and morphological comparison of shallow- and deep-water populations of the introduced dreissenid bivalve *Dreissena bugensis* . Can J Zool 76:1269–1276.

[80] Peyer SM, Hermanson JC, Lee CE (2010) Developmental plasticity of shell morphology of quagga mussels from shallow and deep-water habitats of the Great Lakes. J Exp Biol 213:2602–2609. 20639421 10.1242/jeb.042549

[81] Conn DB, Conn DA (1993) Parasitism, predation and other biotic associations between dreissenid mussels and native animals in the St. Lawrence River. In: Tsou JLeditor. Third International Zebra Mussel Conference. Toronto, ON: Electric Power Research Institute, Paolo Alto, CA. pp. 223–234.

[82] Silayeva AA, Protasov AA (2005) The structure of *Dreissena* communities in the littoral zone of the Kanev reservoir. Vestn Tûmensk Univ 5:112–115.

[83] Casper AF, Johnson LE (2010) Contrasting shell/tissue characteristics of *Dreissena polymorpha* and *Dreissena bugensis* in relation to environmental heterogeneity in the St. Lawrence River. J Great Lakes Res 36:184–189.

[84] Protasov AA, Afanasjev SA, Sinicyna OO, Zdanowski B (1994) Composition and functioning of benthic communities. Arch Pol Fish 2:257–284.

[85] Diggins TP, Weimer M, Stewart KM, Baier RE, Meyer AE, et al (2004) Epiphytic refugium: are two species of invading freshwater bivalves partitioning spatial resources? Biol Invasions 6:83–88.

[86] Ricciardi A, Snyder FL, Kelch DO, Reiswig HM (1995) Lethal and sublethal effects of sponge overgrowth on introduced dreissenid mussels in the Great Lakes - St. Lawrence River System. Can J Fish Aquat Sci 52:2695–2703.

[87] Karatayev AY, Burlakova LE, Padilla DK (1998) Physical factors that limit the distribution and abundance of *Dreissena polymorpha* (Pall.). J Shellfish Res 17:1219–1235.

[88] Lavorel S, Garnier E (2002) Predicting changes in community composition and ecosystem functioning from plant traits: revisiting the Holy Grail. Funct Ecol 16:545–556.

[89] McGill BJ, Enquist BJ, Weiher E, Westoby M (2006) Rebuilding community ecology from functional traits. Trends Ecol Evol 21:178–185. 16701083 10.1016/j.tree.2006.02.002

[90] Flynn DFB, Mirotchnick N, Jain M, Palmer MI, Naeem S (2011) Functional and phylogenetic diversity as predictors of biodiversity-ecosystem-function relationships. Ecology 92:1573–1581. 21905424 10.1890/10-1245.1

[91] Koehl MAR (1996) When does morphology matter? Annu Rev Ecol Syst 27:501–542.

[92] Hayes KR (2003) Biosecurity and the role of risk assessment. In: Ruiz GM, Carlton JTeditors. Invasive Species: Vectors and Management Strategies. Washington, D.C.: Island Press. pp. 382–414.

[93] Keller RP, Drake JM, Lodge DM (2007) Fecundity as a basis for risk assessment of nonindigenous freshwater molluscs. Conserv Biol 21:191–200. 17298525 10.1111/j.1523-1739.2006.00563.x

[94] Ward JM, Ricciardi A (2007) Impacts of *Dreissena* invasions on benthic macroinvertebrate communities: a meta-analysis. Divers Distrib 13:155–165.

[95] Higgins SN, Vander Zanden MJ (2010) What a difference a species makes: a meta-analysis of dreissenid mussel impacts on freshwater ecosystems. Ecol Monogr 80:179–196.

[96] Crail TD, Krebs RA, Zanatta DT (2011) Unionid mussels from nearshore zones of Lake Erie. J Great Lakes Res 37:199–202.

[97] Strayer DL, Smith LC (1996) Relationships between zebra mussels (*Dreissena polymorpha*) and unionid clams during the early stages of the zebra mussel invasion of the Hudson River. Freshw Biol 36:771–779.

[98] Karatayev AY, Burlakova LE, Karatayev VA, Boltovskoy D (2010) *Limnoperna fortunei* versus *Dreissena polymorpha*: population densities and benthic community impacts of two invasive freshwater bivalves. J Shellfish Res 29:975–984.

[99] Mills EL, Leach JH, Carlton JT, Secor CL (1993) Exotic species in the Great Lakes - a history of biotic crises and anthropogenic introductions. J Great Lakes Res 19:1–54.

[100] Mills EL, Casselman JM, Dermott R, Fitzsimons JD, Gal G, et al (2003) Lake Ontario: food web dynamics in a changing ecosystem (1970–2000). Can J Fish Aquat Sci 60:471–490.

[101] Hollandsworth D, Lowe R, Badra P (2011) Indigenous unionid clam refugia from zebra mussels in Michigan inland lakes. Am Midl Nat 166:369–378.

[102] Griffiths RW, Schloesser DW, Leach JH, Kovalak WP (1991) Distribution and dispersal of the zebra mussel (*Dreissena polymorpha*) in the Great Lakes region. Can J Fish Aquat Sci 48:1381–1388.

